# Genetic diversity for drought and low-phosphorus tolerance in rice (*Oryza sativa* L.) varieties and donors adapted to rainfed drought-prone ecologies

**DOI:** 10.1038/s41598-021-93325-2

**Published:** 2021-07-01

**Authors:** Somnath Roy, B. C. Verma, Amrita Banerjee, J. Kumar, Uday Sankar Ray, N. P. Mandal

**Affiliations:** 1 Central Rainfed Upland Rice Research Station, ICAR-National Rice Research Institute, Hazaribag, 825301 Jharkhand India; 2 Zonal Drought Resistant Paddy Research Station, Hathwara, Purulia, 723147 West Bengal India

**Keywords:** Molecular evolution, Natural hazards

## Abstract

Drought and phosphate availability are two major abiotic factors limiting productivity of rice in rainfed upland areas. There has been a constant need for new improved donor with tolerance to multiple abiotic stress conditions for rainfed rice breeding. In the present study, a set of 32 popular rice varieties and landraces were evaluated for drought and low-phosphorus (P) tolerance, and also characterized using grain yield under reproductive drought QTLs (DTY QTLs) and *Pup1* linked/specific molecular markers. Twenty-seven genotypes were identified as tolerant to moderately tolerant to drought. The SSR markers linked to ten DTY QTLs classified the genotypes into two groups corresponding to *aus* and *indica*. The tolerant genotypes were distributed under both groups. Based on the core markers of *Pup1* locus, complete tolerant haplotype was recorded in nine genotypes other than the tolerant check Dular. Nine more genotypes showed the incomplete tolerant haplotypes. The rice genotypes showed significantly high genetic variability for low-P tolerance in hydroponic study. A few genotypes revealed non-*Pup1* type tolerance which needs further confirmation.

## Introduction

Rice (*Oryza sativa* L.) is unique in its ability to grow in wide hydrological environments ranging from aerobic to deep-water situations. Globally, around 40% of rice is produced under rainfed environments^1^. The rainfed rice production environments, and the needs and preferences of the rural people who subsist in them, are highly diverse. Abiotic stress conditions such as water stress, including deficit or drought and excess water or flood, salinity, phosphorus deficiency in soils and heat, cause extensive losses to agricultural production under rainfed areas worldwide^2^.

Of all the abiotic stresses that restrain rice productivity, drought is the most devastating one, particularly in rainfed areas. In India, a recent aspect of southwest monsoon is that some areas of the country such as central, eastern, and northeastern regions are facing the spectra of drought^3^. Severe drought in 2002 and 2009 caused a 20% and 16% reduction in food grain, respectively, leading to higher prices and food security concerns^4^. Therefore, drought-tolerant rice varieties are crucial for food security under situations of accelerating food demand, depleting resources, and high climatic variability^5^. Identifying rice varieties with genes or quantitative trait loci (QTLs) that contribute to stress tolerance and locating such genetic factors on molecular linkage maps offer a precise way to produce stress-tolerant rice varieties via marker-assisted selection (MAS). For drought tolerance at reproductive state, the genetic mapping studies at International Rice Research Institute (IRRI), Philippines has led to the identification of major DTY QTLs (*qDTY*_*1.1*_*–qDTY*_*12.1*_) conditioning higher grain yield under reproductive stage drought tolerance in the background of high yielding varieties: IR64, MTU1010, Swarna, Sabitri, TDK1, and Vandana^6,7^.

Phosphorus (P) is an essential element for all living organisms. Low soil P stress is one of the major constraints on plant growth and yield worldwide in many crops including rice^8,9^. Applying more P fertilizer to mitigate P deficiency increases production costs and import demands and also leads to pollution of water bodies due to fertilizer runoff. Hence, enhancing the P-deficiency tolerance of rice varieties is the cost-effective solution than relying on fertilizer application. Identification of *Phosphorus Uptake 1* (*Pup1*) which is associated with increased P uptake and a significant yield advantage under P-deficient environments^10,11^. *Pup1* was identified from the donor variety Kasalath as a major QTL on rice (*Oryza sativa*) chromosome 12 explaining around 70% of the phenotypic variance for tolerance to phosphorus deficiency in soil^10,12^. Molecular markers were designed for different candidate genes within the *Pup1* locus and effectively been used for detecting *Pup1* haplotypes in diverse rice genotypes^11,13^. The *Pup1* protein kinase gene, *OsPupK46-2,* was functionally characterized and named as *PSTOL1* (*Phosphorus Starvation Tolerance 1*)^14^. Interestingly, *PSTOL1* was found to be absent from the rice reference genome (Nipponbare, a temperate japonica variety), where it falls within a ~ 90 kb insertion-deletion polymorphism (indel) on chromosome 12. *PSTOL1* haplotype analysis in *Oryza* gene pool suggested a long-term selective maintenance of functional alleles, but with repeated evolution of loss-of-function alleles that have evolved convergently in multiple rice species and cultivated rice varieties^15^.

Identifying new donors for abiotic stress tolerance with desirable background is essential for rice breeding under rainfed environments. The donors of many of the DTY QTLs were landraces such as Aday Sel., Dhagad Deshi, Kali aus, Moroberekan, N22, etc.^7^ Similarly, the *Pup1* locus was found in more that 80% of the drought tolerant varieties and breeding lines^11^. In this context, the present study was undertaken using grain yield under drought stress (DTY) QTL-linked SSR and *Pup1-*gene-based markers to assess the diversity based on DTY-QTL makers, and to evaluate *Pup1* haplotypes locus in a diverse set of released varieties and landraces mostly adapted to rainfed drought-prone ecologies. The knowledge of the extent of genetic variation, diversity and presence of stress tolerance genes will lead to exploit the traits for rainfed rice improvement.

## Results

### Genetic diversity analysis based on SSR markers linked to DTY QTLs

Multiple alleles were observed for each of the DTY QTL-linked SSR markers (Supplementary Table S1). A total of 88 alleles were detected at the 24 SSR markers across 32 genotypes, ranging from two alleles (RM28199, *qDTY*_*12.1*_) up to five alleles in RM262 (*qDTY*_*2.1*_), RM211 (*qDTY*_*2.2*_) and RM204 (*qDTY*_*6.1*_), with an average of 3.67 alleles. The polymorphism information content (PIC) ranged from 0.27 (RM279) to 0.70 (RM262) with an average PIC of 0.54. The mean expected heterozygosity (He) was 0.60. The most common allele at each locus ranged from 34% (RM262) to 84% (RM279).

**Figure 1:**
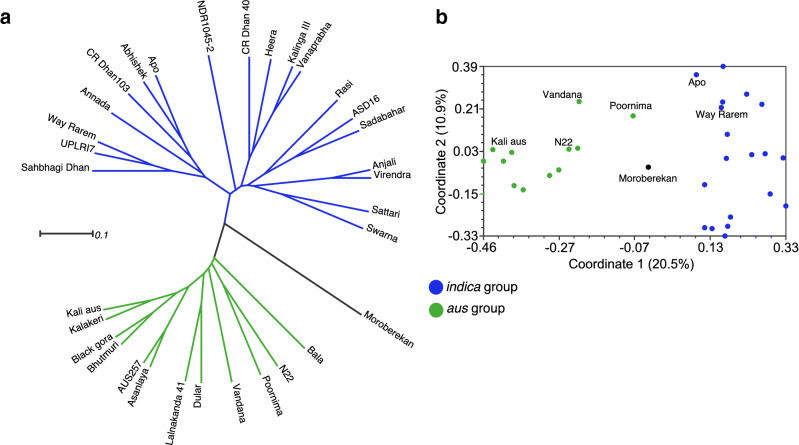
The genetic relationships between 32 rice genotypes based on 24 DTY QTL-linked SSR markers. (**a**) A UPGMA phylogenetic tree showing the clustering of rice cultivars into *indica* and aus groups. (**b**) Principal coordinate analysis with the indica and aus groups colour coded. The figure was edited for presentation using Affinity Designer 1.9.3 (https://affinity.serif.com/en-gb/).

The distance-based results using UPGMA clustering revealed two major groups in the present rice germplasm (Fig. 1a). Twelve genotypes including the known *aus* cultivars and three released varieties (Bala, Poornima and Vandana) corresponds to *aus* group, while 19 *indica* varieties correspond to the *indica* group. The *tropical japonica* cultivar Moroberekan cluster separately from both *indica* and *aus* groups. Similarly, the principal coordinate analysis revealed a clear separation between *indica* and *aus*, although Vandana and Poornima showed some distinctiveness from the primary *aus* cluster (Fig. 1b). The lowest genetic distance was observed between Anjali-Virendra and AUS257-Asanlaya, followed by Kalinga III—Vanaprabha. The highest distance was recorded between Kali aus—Swarna, Bhutmuri—Rasi and Bhutmuri—Sattari.

### Drought tolerance of the studied genotypes

**Figure 2:**
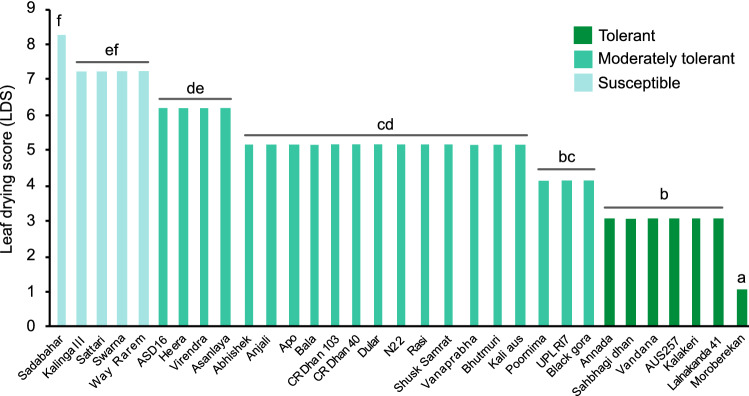
Drought sensitivity in terms of leaf drying score of 32 rice cultivars. Different letters on the bars indicate statistically significant difference as determined by DMRT analysis. The figure was edited for presentation using Affinity Designer 1.9.3 (https://affinity.serif.com/en-gb/).

Significant variation (*P* < 0.000) for leaf drying score (LDS) was observed in 32 rice genotypes (Fig. 2). The lowest LDS was recorded by Moroberekan. Considerable drought tolerance in terms of LDS was observed in six genotypes such as Vandana, Sahbhagi Dhan, Annada, Lalnakanda 41, AUS257 and Kalakeri, and these genotypes were noted as tolerant. The lowest drought tolerance (LDS = 7–9) was exhibited by Sadabahar, followed by Sattari, Kalinga III, Way Rarem and Swarna. Known drought tolerant genotypes such as Black gora, Bhutmuri, Dular, Kali aus and N22 exhibited moderate tolerant with LDS values (4–5).

### Molecular survey with Pup1 markers

**Figure 3:**
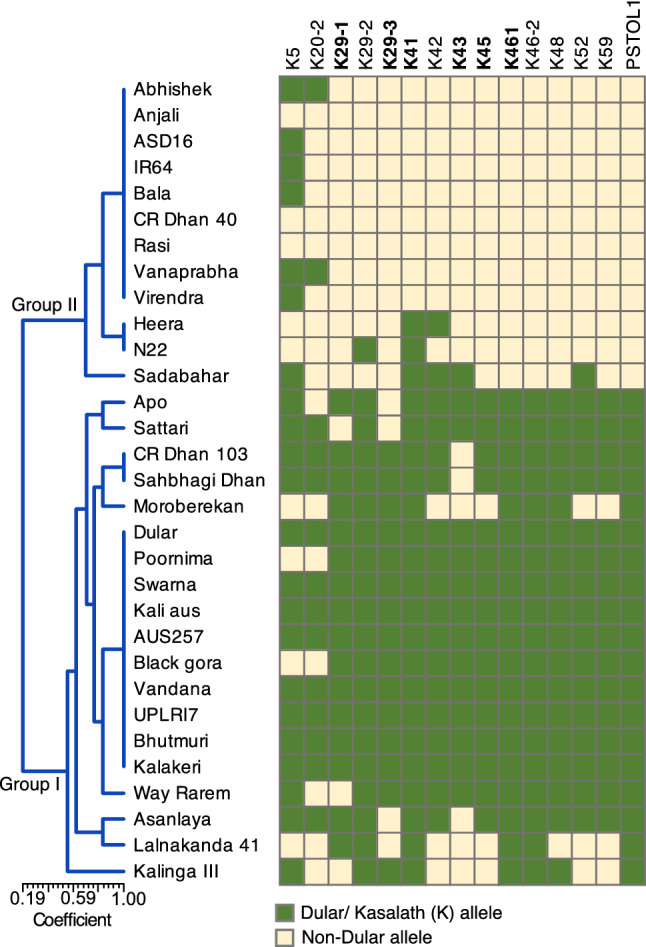
*Pup1* haplotypes in 31 rice genotypes. The core *Pup1* marker set^13^ is highlighted in bold faces letters. An UPGMA cluster analysis was conducted using simple matching similarity coefficient based on core markers in NTSYS-pc. *Pup1* positive check is Dular which is reported to possess all *Pup1* makers. The Dular/ Kasalath and non-Dular alleles are colour coded. The figure was edited for presentation using Affinity Designer 1.9.3 (https://affinity.serif.com/en-gb/).

The *Pup1* haplotypes were detected in 31 genotypes (including sensitive check IR64) using 14 *Pup1* specific markers (Fig. 3). Six genotypes such as Vandana, AUS257, Kalakeri, Kali aus, Bhutmuri, and UPLRI7 possess Dular allele at all 14 loci. The *Pup1* tolerant check Dular possesses Kasalath (K) allele or tolerant allele at all *Pup1* marker loci^13^. Among the genotypes, the K allele frequency varied from 45% (K20-2 and K43) to 70% (K5 and K41). A higher frequency was also recorded for K29-2 (64%), K46-1 (61%) and K46-2 (61%). A few genotypes such as Sahbhagi Dhan, Apo, CR Dhan 103, Swarna, Black gora, Poornima, Way Rarem and Asanlaya showed K allele for 12–13 *Pup1* markers. Cluster analysis using six core *Pup1* markers data classified the genotypes into two major groups. Group I included 19 genotypes (including Dular) with all or most K *Pup1* alleles (Fig. 3). The genotypes in group II did not possess any K allele except N22 and Sadabahar which showed the presence of K41, and K41 and K43, respectively. In the present study the data obtained with two *Pup1* protein kinase gene specific markers, K46-1 and K46-2, were consistent and both the markers were detected in the same 19 genotypes. Subsequently, a survey with *PSTOL1* gene specific marker also revealed similar results for the presence of the protein kinase gene (Fig. 3). Note that, all these 19 genotypes are represented in group I.

### Phenotypic evaluation for low P tolerance

**Figure 4:**
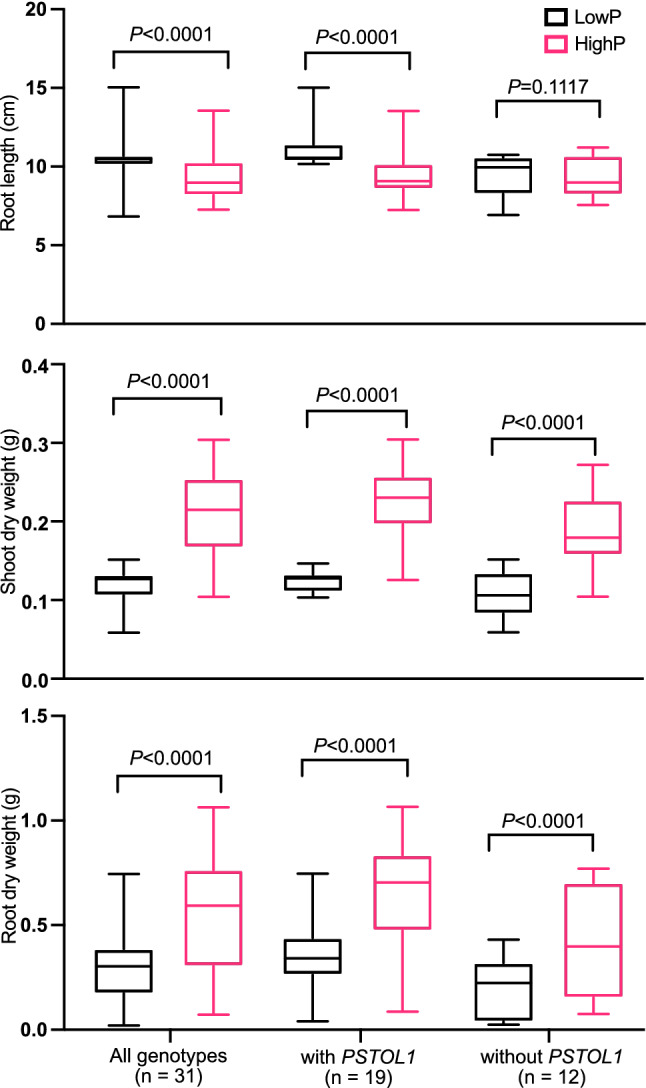
Performance of rice genotypes under two phosphorus conditions for three different growth traits. The figure was edited for presentation using Affinity Designer 1.9.3 (https://affinity.serif.com/en-gb/).

**Figure 5:**
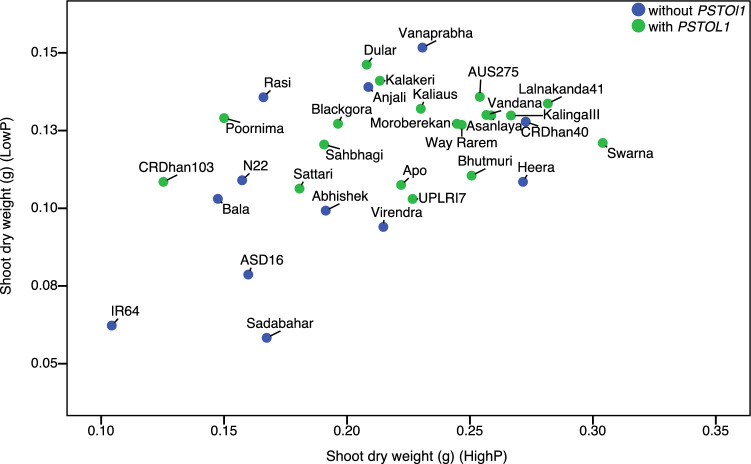
Relationship of shoot dry weight in low- and high P treatments. Genotypes in the upper right quadrant are vigorous under both conditions. The figure was edited for presentation using Affinity Designer 1.9.3 (https://affinity.serif.com/en-gb/).

The low phosphorus (P) treatment was effective in producing P deficiency stress, as shown by reduced shoot biomass. Significant differences among the genotypes were observed for all three root and shoot traits studied. Additionally, the interaction between genotype and P treatment was significant (*P* < 0.0001) indicating that the impact of P levels on the growth traits depends on genotype (see Supplementary Table S2 online). Under low P conditions, root length increased significantly. However, root length did not show significant differences between high- and low P conditions in plants lacking the *PSTOL1* gene (Fig. 4). In contrast, the plants with *PSTOL1* produced longer roots under P deficient conditions. Both shoot and root growth reduced significantly under low P treatment regardless of presence or absence of *PSTOL1* (Fig. 4). Root length ranged from 7.3 cm (Kalinga III) to 13.5 cm (Moroberekan) under high P, whereas under low P it varied between 6.8 cm (IR64) to 15.1 cm (Moroberekan). The root length increased up to 41.1% (Kalinga III) with an average of 12.2% with the genotypes under low P. Overall, seven genotypes did not show longer roots under P deficiency (see Supplementary Fig. S1 online). The mean shoot dry weight of *PSTOL1* genotypes was similar to that of the genotypes without *PSTOL1* under P deficient conditions. However, under high P conditions, root dry weight was considerably higher in genotypes carrying *PSTOL1*. The shoot dry weights under low P against those under high P was plotted to identify genotypes with high vigour under both P levels (Fig. 5). There was a wide variation in vigour among the genotypes. Swarna, Lalnakanda 41, Vandana, Asanlaya, AUS257, CR Dhan 40, Kalinga III and Moroberekan were the most vigorous genotypes under both P treatments.

## Discussion

The DTY QTLs have a genetic gain of 10–30%, with a yield advantage of 150–500 kg ha^−1^ under reproductive stage moisture stress^16^. Therefore, improvement of rice yield under drought targeted through pyramiding genetically compatible major DTY QTLs in the background of popular rice varieties^6,17–21^. For the breeders, only the QTLs that express in a range of genetic backgrounds and environments are of interest and can be deployed widely for developing drought tolerant rice varieties. Seven DTY QTLs: *qDTY*_*1.1*_*, qDTY*_*2.2*_*, qDTY*_*3.1*_*, qDTY*_*3.2*_*, qDTY*_*4.1*_*, qDTY*_*6.1*_, and *qDTY*_*12.1*_ have shown large effects across two or more genetic backgrounds and under both transplanted lowland and direct seeded upland environments^5^. Moreover, identification of new QTL donors with desirable background using molecular markers tightly linked to DTY QTLs could be an attractive option. In recent year, a couple of studies have been published in this direction^22,23^. Although such studies can only predict the presence/ absence of a QTL in a new genotypes, these have some importance for identification of novel tolerant QTLs by involving the germplasm lines without any of the reported DTY QTLs. In the present study, multiple alleles (average 3.67 alleles/locus) have been detected for the 24 DTY QTL-linked SSR markers, which indicate non-conservativeness of drought tolerant QTLs in rice.

When the banding patterns of the present genotypes were compared with the original donor of any DTY QTL, it was revealed that Asanlaya, AUS 257, Bhutmuri, Kalakeri, Dular and Lalnakanda 41 had similar alleles to kali aus at all the linked SSR markers of *qDTY*_*1.2*_ and *qDTY*_*1.3*_ (see Supplementary Table S3 online). Similarly, *qDTY*_*2.2*_ specific banding was obtained in Kalakeri, AUS 257 and Black gora; *qDTY*_*2.1*_ in CR Dhan 103 and Sahbhagi Dhan; *qDTY*_*3.2*_ in Kalakeri, Poornima and Vandana; *qDTY*_*6.1*_ in Abhishek, Apo, Kalakeri and Poornima; and *qDTY*_*12.1*_ in Sahbhagi Dhan and UPLRI7. Note that, the drought tolerant variety Vandana was developed from the cross C22/Kalakeri, and at least two drought QTLs (*qDTY*_*2.3*_ and *qDTY*_*6.1*_) of Vandana were transmitted from Kalakaeri^24^. The present analysis also indicates that Kalakeri, a highly drought tolerant landrace from the Indian state of Odisha, could be utilized further in drought breeding programmes. Likewise, other *aus* cultivars such as Black gora, Bhutmuri, Asanlaya, Lalnakanda 41 and AUS 257 clould also be utilized for identification of novel QTLs for drought tolerance.

Genetic diversity analysis of the 32 rice genotypes using the drought QTL-linked SSR markers revealed two major groups, and based on the previous reports, we could designate one group as *indica* and the other as *aus*. The *aus* group included all drought tolerant *aus* cultivars along with the varieties having *aus* in their parentage. Earlier reports also indicated a common ancestry of drought-tolerant rice genotypes, mostly from the *aus* group^25^. In the present study, the DTY QTL markers effectively revealed the ancestry and genetic differentiation within the genotype panel.

For detection of tolerance alleles at the *Pup1* locus in the present genotypes, the genotypes along with low P tolerant and sensitive checks were surveyed with 14 gene-specific markers. The frequency of different *Pup1* genes varied widely across the genotypes. *Pup1* is a major locus on rice chromosome 12 conferring tolerance to P deficiency in soils. This locus contributed around 70% of the total phenotypic variance for P uptake^10,12^. This locus is prevalent in varieties and landraces adapted to rainfed drought-prone and acidic soil environments^13,26^. Our study also indicated a similar trend that *Pup1* is present in most of the drought tolerant genotypes either completely or partially. Therefore, it is safe to note that, the breeders have directly or indirectly selected for the *Pup1* QTL while developing resilient varieties for the rainfed environments. A cluster analysis using six core *Pup1* markers grouped the genotypes based on the presence or absence of respective *Pup1* marker loci and provided details on the different haplotypes present in the current genotypes. Altogether, ten genotypes (including tolerant check Dular) had the Kasalath allele at all six loci, and these genotypes along with nine others possessing the tolerant haplotype partially formed a separate cluster from the genotypes that are completely devoid of the Kasalath haplotypes. We got a variable result with the three markers targeting the hypothetical gene *OsPupK29*. The frequency of tolerant allele these marker loci varied from 48% (K29-3) to 64% (K29-2). Chin et al.^13^ reported that *OsPupK29* is an unstable gene with possible role in the evolution of *Pup1*. The protein kinase gene *OsPupK46* was characterized as the candidate gene *PSTOL1* underlying the *Pup1* locus^14^. Genotyping with the markers specific to *PSTOL1* and *OsPupK46* revealed a similar result with a frequency of 61% in the current genotypes. The tolerant genotypes identified in this study with the null *Pup1* will be utilized in future investigations on the low P tolerance mechanisms. Swamy et al.^26^ also reported null *Pup1* tolerant genotypes in rice collections from north-eastern India.

Phenotypic evaluation of the genotypes under low- and high P conditions revealed both genetic variation in root and shoot traits and the genotypic response to P deficiency. When shoot dry weight under low P was plotted as a function of that of high P, a wide variation in plant vigour was observed. Genotypes such as Swarna, Kalinga III, Vanaprabha, Heera, Way Rarem, Vandana, CR Dhan 40, and the *aus* cultivars were vigorous under both P levels. Among these genotypes a few (Rasi, Vanaprabha and CR Dhan 40) were devoid of the tolerant *Pup1* haplotype suggesting a non-*Pup1* type tolerance. However, the result needs to be revalidated under field evaluation. The *aus* cultivars showed considerable *Pup1* type tolerance to low P. The *aus* genotypes are well recognized as a source of abiotic stress tolerance in rice breeding^27–29^.

In conclusion, the present study had characterized a set of popular rainfed rice varieties and landraces for tolerance to two most important abiotic stresses, drought and low P, widespread under rainfed drought-prone ecologies in India. The results of genotyping with DTY QTL-linked and *Pup1-*specific markers will help rice breeders in selecting suitable parents for varietal development.

## Methods

### Study site and plant materials

The study was conducted at the experimental facilities of Central Rainfed Upland Rice Research Station (CRURRS), ICAR-National Rice Research Institute, Hazaribag, Jharkhand (23°57′35″ N latitude, 85°22′12″ E longitude and 610 m altitude). The soil of the field is an acid alfisol with a pH of 5.2, organic carbon 4.68 g kg^−1^, available N 278.0 kg ha^−1^ and P 9.88 kg ha^−1^. A total of 32 rice genotypes comprising of rice varieties and landraces mostly of rainfed drought-prone ecologies, were taken from the rice genebank of ICAR-National Rice Research Institute, Cuttack (India). The list of genotypes can be found as Supplementary Table S4 online. The genotypes Apo, Kali aus, Moroberekan, N22, Vandana and Way Rarem were reported donors of several DTY QTLs^6^. The variety Dular was used as positive check for *Phosphorus uptake 1* (*Pup1*) survey. It had Kasalath-type alleles for all the *Pup1* genes^13^. The experiments on these plant materials were done as a part of inhouse research project of ICAR-National Rice Research Institute for developing resilient rice varieties for rainfed drought-prone ecologies.

### Drought screening

The rice genotypes were screened under rainout shelter screening facility at CRURRS for drought tolerance during August-October 2018 wet season as per the protocol described in Verulkar et al.^30^ The genotypes were dry seeded under upland conditions in rows spaced at 25 cm in well prepared levelled fields. The experiment was conducted in a randomized complete block design with three replications. Inorganic NPK fertilizer dose of 60–40–40 was applied. P and K were applied as a single basal dose at sowing, whereas N was applied in three equal splits, at sowing, 28 and 50 days after sowing. Weeds were controlled by application of the post-emergence herbicide bispyribac sodium (0.02 kg a.i. ha^−1^) at five days after sowing. Two hand weedings were done at later stages to keep the plots weed-free. The crop was flood-irrigated twice a week until 50 DAS. At 50 DAS, stress was imposed by stopping irrigation and protecting the trial from rainfall by covering it with a shelter during rainfall events. Genotypes were scored for leaf drying at the peak stress period (about three weeks after the withdrawal of irrigation) using the IRRI Standard Evaluation System^31^. With appearance of severe leaf rolling and drying on the susceptible check (Kalinga III), at about 21 days after withdrawal of irrigation, the stress was relieved by one flood irrigation. A second cycle of stress was then imposed until the maturity of the crop.

### Low P screening

Sensitivity of the genotypes to P-deficient growing conditions was measured by root growth and shoot mass under low- and high P treatments under hydroponic experiments in the greenhouse. The seeds were surface sterilized with 1% sodium hypochlorite solution for 1 h with agitation and then rinsed five time with sterile doubled distilled water. Surface sterilized seeds were then sown on moist paper towel soaked with sterile water for 72 h at 28 °C in an incubator prior to planting. Healthy germinated seeds were then individually placed in the holes (1.5–2.0 cm in diameter) made on a Styrofoam sheet (50 cm × 60 cm) floated in a plastic tank containing 40 L tap water. A total of 96 holes were made per sheet. The experiment was laid out in a completely randomized design with three replications. In each replication, three germinated seeds of each variety were sown in three adjacent holes. After seven days, the seedlings were evaluated in two hydroponic solutions, one with deficient P (LowP) and the other with sufficient P (HighP). Both Low P and High P conditions were established by maintaining the NaH_2_PO_4_ concentration in the hydroponic media^32^ as 10 µM and 100 µM, respectively. The nutrient solution was changed every 3 days and maintained at a pH of 5.0 by adding 1 M NaOH or HCl as needed. The plants were sampled at 14 days after treatment. The root length of each plant was recorded. Plants were then partitioned into shoots and roots and dried in an oven at 70 °C for 72 h and weighed. Rice genotypes Annada and NDR 1045-2 were not included due to nonavailability of pure seeds.

### Genotyping with DTY QTL and Pup1 markers

Twenty-four SSR markers were selected for detection of the DTY QTLs (Table 1). The peak and flanking markers for each DTY QTLs were included based on the information summarized in Kumar et al.^6^. For genotyping of *Pup1* locus and *PSTOL1,* 15 gene-specific markers were used (Table 1). Positive checks were included in both the assays.

**Table 1:** Markers used in the study.

QTL/gene	Chromosome	Marker(s)	Donor/positive check	Reference
**DTY QTLs**				
*qDTY*_*1.1*_	1	RM11943, RM431, RM12091	N22	^35^
*qDTY *_*1.2*_	1	RM259, RM315	Kali aus	^36^
*qDTY *_*1.3*_	1	RM488, RM315	Kali aus	^36^
*qDTY *_*2.1*_	2	RM327, RM262	Apo	^37^
*qDTY *_*2.2*_	2	RM211, RM263, RM279, RM555	Kali aus	^36^
*qDTY *_*2.3*_	2	RM263, RM573, RM3212, RM250	Kali aus	^36,38^
*qDTY *_*3.1*_	3	RM520, RM416	Apo	^37^
*qDTY *_*3.2*_	3	RM60, RM22	N22	^35^
*qDTY *_*6.1*_	6	RM589, RM204	Vandana	^39^
*qDTY *_*12.1*_	12	RM28048, RM28166, RM28199	Way rarem	^40^
**Pup1 candidate genes**				
*OsPupK05-1*	12	K5 (co-dominant)	Dular^a^	^13^
*OsPupK20-2*	12	K20-2 (co-dominant)	Dular	^13^
*OsPupK29-1*	12	K29-1 (co-dominant)	Dular	^13^
*OsPupK29-1*	12	K29-2 (co-dominant)	Dular	^13^
*OsPupK29-1*	12	K29-3 (co-dominant)	Dular	^13^
*OsPupK41-1*	12	K41 (dominant)	Dular	^13^
*OsPupK42-1*	12	K42 (dominant)	Dular	^13^
*OsPupK43-1*	12	K43 (dominant)	Dular	^13^
*OsPupK45-1*	12	K45 (dominant)	Dular	^13^
*OsPupK46-2*	12	K46-1 (dominant)	Dular	^13^
*OsPupK46-2*	12	K46-2 (dominant)	Dular	^13^
*OsPupK48-1*	12	K48 (dominant)	Dular	^13^
*OsPupK52-1*	12	K52 (dominant)	Dular	^13^
*OsPupK59-1*	12	K59 (dominant)	Dular	^13^
*PSTOL1*	12	PSTOL1 (dominant)	Dular	^14^

^a^Dular has been taken as positive check in the present study for *Pup1* genotyping. Dular and Kasalath (original donor of *Pup1*) had similar alleles for all the gene models at *Pup1* locus Chin et al.^13^.

The total genomic DNA was extracted from the ground leaf tissue using Qiagen DNeasy Pant Mini Kit (Qiagen, India). The extracted DNA was quantified using a UV–Vis spectrophotometer (NanoDrop 2000c, Thermo Scientific, USA) and was adjusted to a final concentration of 25 ng µL^−1^. Polymerase chain reaction (PCR) was carried out in a reaction mix containing 50 ng template DNA, 0.5 μM of each primer, 12.5 μL PCR Master Mix (2X) (Thermo Scientific, USA) and double-distilled water to make the total volume up to 25 μL. Amplification was carried out using thermal cycler with the following thermal conditions: 94 °C for 5 min, followed by 35 cycles of 94 °C for 30 s, primer-specific annealing temperature for 30 s, 72 °C for 1 min, and final extension step was included for 72 °C for 7 min. The amplified products were visualized by SYBR™ Safe DNA Gel (Thermo Scientific, USA) stained agarose gels (1.0–4.0% based on amplicon size) in gel documentation system. In case of DTY QTL-linked SSR markers, the band with the lowest molecular weight was assigned allele number 1 and the progressively heavier bands were assigned numbers incrementally. For *Pup1*-specific markers, the molecular sizes (in base pair) of the amplified alleles were determined based on migration relative to the DNA ladder. The reproducible and well amplified DNA fragments were considered for analysis.

### Data analysis

The drought and P-deficient screening data were subjected to the statistical analysis in R version 3.5.2 (The R Foundation for Statistical Computing, Vienna, Austria) using the packages “tidyverse” and “rstatix”. One- and Two-way analysis of variance (ANOVA) were used to examine the influence of treatments on dependant variables and interactions between genotype and treatment level. The mean leaf drying scores of 32 genotypes were compared by the Duncan's Multiple Range Test (DMRT).

The number of alleles locus^−1^, major allele frequency, gene diversity, and polymorphism information content (PIC) were estimated using PowerMarker V3.25^33^. The genetic distance between all possible pairs of accessions were calculated and used to visualize relationships between accessions, and a phylogenetic tree was constructed using neighbour joining clustering using the C.S. Chord 1967 distance method implemented in PowerMarker V3.25. The allele frequency data from PowerMarker was used to export the data in binary format (allele presence = 1, and allele absence = 0) for analysis with NTSYS-pc V2.2^34^. A similarity matrix was calculated with the SIMQUAL subprogram using the Jaccard’s coefficient and was also used for principal coordinate analysis with the DCENTER, EIGEN and MXPLOT subprograms in NTSYS-pc.

## Supplementary Information

Supplementary Information.

## Data Availability

Available on request from the authors.

## References

[1] Singh K, McClean CJ, Büker P, Hartley SE, Hill JK (2017). Mapping regional risks from climate change for rainfed rice cultivation in India. Agric. Syst..

[2] Haefele SM, Nelson A, Hijmans RJ (2014). Soil quality and constraints in global rice production. Geoderma.

[3] Tyalagadi MS, Gadgil A, Krishnakumar G (2015). Monsoonal droughts in India: A recent assessment. Pap. Glob. Chang. IGBP.

[4] Pandey, S. & Bhandari, H. Drought: Economic costs and research implications. in *Drought Frontiers in Rice: Crop Improvement for Increased Rainfed Production *(eds. Serraj, J., Bennet, J., & Hardy, B.) 3–17 (World Scientific Publishing, 2009).

[5] Sandhu N (2018). Positive interactions of major-effect QTLs with genetic background that enhances rice yield under drought. Sci. Rep..

[6] Kumar A (2014). Breeding high-yielding drought-tolerant rice: Genetic variations and conventional and molecular approaches. J. Exp. Bot..

[7] Sandhu N, Kumar A (2017). Bridging the rice yield gaps under drought: QTLs, genes, and their use in breeding programs. Agronomy.

[8] Dobermann A, White PF (1999). Strategies for nutrient management in irrigated and rainfed lowland rice systems. Nutr. Cycl. Agroecosyst..

[9] Zhang D (2014). The acid phosphatase-encoding gene GmACP1 contributes to soybean tolerance to low-phosphorus stress. PLoS Genet..

[10] Wissuwa M, Yano M, Ae N (1998). Mapping of QTLs for phosphorus-deficiency tolerance in rice (*Oryza sativa* L.). Theor. Appl. Genet..

[11] Chin JH (2010). Development and application of gene-based markers for the major rice QTL *Pphosphorus uptake 1*. Theor. Appl. Genet..

[12] Wissuwa M, Wegner J, Ae N, Yano M (2002). Substitution mapping of Pup1: A major QTL increasing phosphorus uptake of rice from a phosphorus-deficient soil. Theor. Appl. Genet..

[13] Chin JH (2011). Developing rice with high yield under phosphorus deficiency: *Pup1* sequence to application. Plant Physiol..

[14] Gamuyao R (2012). The protein kinase Pstol1 from traditional rice confers tolerance of phosphorus deficiency. Nature.

[15] Vigueira CC, Small LL, Olsen KM (2016). Long-term balancing selection at the *Phosphorus Starvation Tolerance 1* (*PSTOL1*) locus in wild, domesticated and weedy rice (*Oryza*). BMC Plant Biol..

[16] Swamy BPM, Kumar A (2013). Genomics-based precision breeding approaches to improve drought tolerance in rice. Biotechnol. Adv..

[17] Swamy BPM (2013). Genetic, physiological, and gene expression analyses reveal that multiple QTL enhance yield of rice mega-variety IR64 under drought. PLoS ONE.

[18] Dixit S (2014). QTLs for tolerance of drought and breeding for tolerance of abiotic and biotic stress: An integrated approach. PLoS ONE.

[19] Shamsudin NAA (2016). Marker assisted pyramiding of drought yield QTLs into a popular Malaysian rice cultivar, MR219. BMC Genet..

[20] Sandhu N (2019). Marker assisted breeding to develop multiple stress tolerant varieties for flood and drought prone areas. Rice.

[21] Dhawan G (2021). Introgression of qDTY1.1 governing reproductive stage drought tolerance into an elite basmati rice variety “Pusa Basmati 1” through marker assisted backcross breeding. Agronomy.

[22] Anupam A (2017). Genetic diversity analysis of rice germplasm in Tripura state of Northeast India using drought and blast linked markers. Rice Sci..

[23] Mukherjee M (2018). Revealing genetic relationship and prospecting of novel donors among upland rice genotypes using qDTY-linked SSR markers. Rice Sci..

[24] Dixit S (2012). Increased drought tolerance and wider adaptability of qDTY12.1 conferred by its interaction with qDTY2.3 and qDTY3.2. Mol. Breed..

[25] Vikram P (2016). Genetic diversity analysis reveals importance of Green Revolution gene (*Sd1* locus) for drought tolerance in rice. Agric. Res..

[26] Swamy HKM (2019). Phenotypic and molecular characterization of rice germplasm lines and identification of novel source for low soil phosphorus tolerance in rice. Euphytica.

[27] Gowda VRP, Henry A, Vadez V, Shashidhar HE, Serraj R (2012). Water uptake dynamics under progressive drought stress in diverse accessions of the OryzaSNP panel of rice (*Oryza sativa*). Funct. Plant Biol..

[28] Bin Rahman ANMR, Zhang J (2016). Flood and drought tolerance in rice: Opposite but may coexist. Food Energy Secur..

[29] Vejchasarn P, Lynch JP, Brown KM (2016). Genetic variability in phosphorus responses of rice root phenotypes. Rice.

[30] Verulkar SB (2010). Breeding resilient and productive genotypes adapted to drought-prone rainfed ecosystem of India. Field Crops Res..

[31] International Rice Research Institute (2013). Standard Evaluation System for Rice (SES).

[32] Yoshida S, Forno DA, Cock JH, Gomez KA (1972). Laboratory Manual for Physiological Studies of Rice.

[33] Liu K, Muse SV (2005). PowerMarker: An integrated analysis environment for genetic marker analysis. Bioinformatics.

[34] Rohlf FJ (2007). NTSYS-pc: Numerical Taxonomy System, ver. 2.20.

[35] Vikram P (2011). QDTY1.1, a major QTL for rice grain yield under reproductive-stage drought stress with a consistent effect in multiple elite genetic backgrounds. BMC Genet..

[36] Sandhu N (2014). Identification and mapping of stable QTL with main and epistasis effect on rice grain yield under upland drought stress. BMC Genet..

[37] Venuprasad R (2009). Identification and characterization of large-effect quantitative trait loci for grain yield under lowland drought stress in rice using bulk-segregant analysis. Theor. Appl. Genet..

[38] Palanog AD (2014). Grain yield QTLs with consistent-effect under reproductive-stage drought stress in rice. Field Crops Res..

[39] Venuprasad R (2012). A large-effect QTL for rice grain yield under upland drought stress on chromosome 1. Mol. Breed..

[40] Bernier J, Kumar A, Ramaiah V, Spaner D, Atlin G (2007). A large-effect QTL for grain yield under reproductive-stage drought stress in upland rice. Crop Sci..

