# Supplementary material for: Genetic diversity for drought and low-phosphorus tolerance in rice (Oryza sativa L.) varieties and donors adapted to rainfed drought-prone ecologies
Source: Sci Rep. 2021 Jul 1;11:13671. doi: 10.1038/s41598-021-93325-2 (PMC8249610; doi:10.1038/s41598-021-93325-2)
Supplement: Supplementary file 1 — Supplementary Information. [file 41598_2021_93325_MOESM1_ESM.pdf]

Genetic diversity for drought and low-Phosphorus tolerance in rice (*Oryza sativa* L.) varieties and donors adapted to rainfed drought-prone ecologies

Somnath Roy[1]*, B. C. Verma[1], Amrita Banerjee[1], J. Kumar[1], Uday Sankar Ray[2], N. P. Mandal[1]*

[1]*Central Rainfed Upland Rice Research Station, ICAR-National Rice Research Institute, Hazaribag 825301, Jharkhand, India*
[2]*Zonal Drought Resistant Paddy Research Station, Hathwara, Purulia 723147, West Bengal, India*

*e-mail:
S Roy: somnath.roy@icar.gov.in; sroypbr@gmail.com
NP Mandal: npmandal@hotmail.com

Table S1 Diversity statistics of the DTY QTL-linked SSR markers

#	DTY QTL	Marker	MAF	Na	H_e	PIC
1	*qDTY*$_{1.1}$	RM431	0.44	3	0.63	0.56
2	*qDTY*$_{1.1}$	RM11943	0.41	3	0.66	0.58
3	*qDTY*$_{1.1}$	RM12091	0.50	4	0.66	0.60
4	*qDTY*$_{1.2}$	RM259	0.56	4	0.58	0.51
5	*qDTY*$_{1.2,\ 1.3}$	RM315	0.63	3	0.52	0.45
6	*qDTY*$_{1.3}$	RM488	0.41	3	0.63	0.56
7	*qDTY*$_{2.1}$	RM327	0.41	4	0.67	0.61
8	*qDTY*$_{2.1}$	RM262	0.34	5	0.74	0.70
9	*qDTY*$_{2.2}$	RM211	0.41	5	0.73	0.69
10	*qDTY*$_{2.2,\ 2.3}$	RM263	0.50	4	0.63	0.57
11	*qDTY*$_{2.2}$	RM279	0.84	4	0.28	0.27
12	*qDTY*$_{2.2}$	RM555	0.38	4	0.68	0.61
13	*qDTY*$_{2.3}$	RM573	0.47	4	0.65	0.59
14	*qDTY*$_{2.3}$	RM3212	0.47	4	0.65	0.59
15	*qDTY*$_{2.3}$	RM250	0.41	4	0.67	0.60
16	*qDTY*$_{3.1}$	RM 520	0.38	4	0.70	0.64
17	*qDTY*$_{3.1}$	RM416	0.63	3	0.49	0.40
18	*qDTY*$_{3.2}$	RM 60	0.81	3	0.32	0.29
19	*qDTY*$_{3.2}$	RM22	0.56	3	0.58	0.51
20	*qDTY*$_{6.1}$	RM589	0.38	3	0.66	0.59
21	*qDTY*$_{6.1}$	RM204	0.41	5	0.72	0.68
22	*qDTY*$_{12.1}$	RM28166	0.44	4	0.66	0.60
23	*qDTY*$_{12.1}$	RM28199	0.69	2	0.43	0.34
24	*qDTY*$_{12.1}$	RM28048	0.50	3	0.53	0.42
		**Mean**	**0.50**	**3.67**	**0.60**	**0.54**

MAF, major allele frequency; Na, number of alleles; H_e, gene diversity; PIC, polymorphism information content

Table S2 Analysis of variance for root length, shoot dry weight and root dry weight in lowP and highP experiments in completely randomized design

**Source**	**df**	**MS**	**F (DFn, DFd)**	***P* value**
Root length (all genotype)				
Interaction	30	2.610	F (30, 124) = 9.949	P<0.0001
Genotype	30	9.812	F (30, 124) = 37.41	P<0.0001
Treatment	1	59.42	F (1, 124) = 226.5	P<0.0001
Residual	124	0.2623		
Root length (-PSTOL1 genotype)				
Interaction	11	3.158	F (11, 48) = 11.12	P<0.0001
Genotype	11	7.031	F (11, 48) = 24.75	P<0.0001
Treatment	1	0.7462	F (1, 48) = 2.626	P=0.1117
Residual	48	0.2841		
Root length (+PSTOL1 genotype)				
Interaction	18	1.018	F (18, 76) = 4.096	P<0.0001
Genotype	18	9.662	F (18, 76) = 38.88	P<0.0001
Treatment	1	83.90	F (1, 76) = 337.6	P<0.0001
Residual	76	0.2485		
Shoot dry weight (all genotype)				
Interaction	30	0.002753	F (30, 124) = 8.850	P<0.0001
Genotype	30	0.006077	F (30, 124) = 19.53	P<0.0001
Treatment	1	0.4255	F (1, 124) = 1368	P<0.0001
Residual	124	0.0003111		
Shoot dry weight (-PSTOL1 genotype)				
Interaction	11	0.002685	F (11, 48) = 7.963	P<0.0001
Genotype	11	0.007676	F (11, 48) = 22.76	P<0.0001
Treatment	1	0.1313	F (1, 48) = 389.2	P<0.0001
Residual	48	0.0003372		
Shoot dry weight (+PSTOL1 genotype)				
Interaction	18	0.002775	F (18, 76) = 9.421	P<0.0001
Genotype	18	0.003611	F (18, 76) = 12.26	P<0.0001
Treatment	1	0.2974	F (1, 76) = 1010	P<0.0001
Residual	76	0.0002946		
Root dry weight (all genotype)				
Interaction	30	0.03106	F (30, 124) = 5.153	P<0.0001
Genotype	30	0.2926	F (30, 124) = 48.54	P<0.0001
Treatment	1	3.258	F (1, 124) = 540.4	P<0.0001
Residual	124	0.006028		
Root dry weight (-PSTOL1 genotype)				
Interaction	11	0.02584	F (11, 48) = 7.997	P<0.0001
Genotype	11	0.2326	F (11, 48) = 71.99	P<0.0001
Treatment	1	0.8262	F (1, 48) = 255.8	P<0.0001
Residual	48	0.003230		
Root dry weight (+PSTOL1 genotype)				
Interaction	18	0.03182	F (18, 76) = 4.083	P<0.0001
Genotype	18	0.2605	F (18, 76) = 33.42	P<0.0001
Treatment	1	2.506	F (1, 76) = 321.5	P<0.0001
Residual	76	0.007794		

Table S3 DTY QTL positive genotypes identified through QTL-linked SSR markers

Serial No.	Genotype	$qDTY_{1.1}$	$qDTY_{1.2}$	$qDTY_{1.3}$	$qDTY_{2.1}$	$qDTY_{2.2}$	$qDTY_{2.3}$	$qDTY_{3.1}$	$qDTY_{3.2}$	$qDTY_{6.1}$	$qDTY_{12.1}$
1	Abhishek	-	-	-	-	-	-	-	-	√	-
2	Anjali	-	-	-	-	-	-	-	-	-	
3	Annada	-	-	-	-	-	-	-	-	-	
4	Apo	-	-	-	PC	-	-	PC	-	√	-
5	ASD16	-	-	-	-	-	-	-	-	-	-
6	Bala	-	-	-	-	-	-	-	-	-	-
7	CR Dhan 103	-	-	-	√	-	√	-	-	-	-
8	CR Dhan 40	-	-	-	-	-	-	-	-	-	-
9	Dular	-	√	√							
10	Heera	-	-	-	-	-	-	-	-	-	-
11	Kalinga III	-	-	-	-	-	-	-	-	-	-
12	Nagina 22	PC*	-	-	-	-	-	-	PC	-	-
13	Poornima	-	-	-	-	-	-	-	√	√	
14	Rasi	-	-	-	-	-	-	-	-	-	-
15	Sadabahar	-	-	-	-	-	-	-	-	-	-
16	Sahbhagi Dhan	-	-	-	√	-	-	-	-	-	√
17	Sattari	-	-	-	-	-	-	-	-	-	-
18	Shusk Samrat	-	-	-	-	-	-	-	-	-	-
19	Swarna	-	-	-	-	-	-	-	-	-	-
20	UPLRI7	-	-	-	-	-	-	-	-	-	√
21	Vanaprabha	-	-	-	-	-	-	-	-	-	
22	Vandana	-	-	√	-	-	√	-	√	PC	-
23	Virendra	-	-	-	-	-	-	-	-	-	-
24	Way Rarem	-	-	-	-	-	-	-	-	-	PC
25	Bhutmuri	-	√	√	-	-	-	-	-	-	-
26	AUS 257	-	√	√	-	√	-	-	-	-	-
27	Black gora	-	-	-	-	√		-	-	-	-
28	Kalakeri	-	√	√	-	√	√	-	√	√	-
29	Kali aus	-	PC	PC	-	PC	PC	-	-	-	-
30	Asanlaya	-	√		-	-	-	-	-	-	-
31	Lalnakanda-41	-	√	√	-	-	-	-	-	-	-
32	Moroberekan	-			-	-	-	-	-	-	-

*PC, Positive check for the DTY QTL

Table S4 Details of rice genotypes used in the present study

Serial No.	Name of cultivar	Parentage	Year of Release	Released from	Area of adaptability	Agro-ecology	Salient features[b]
1[a]	Abhishek (RR 272-829)	Natural cross of CR 314-5-10 (mutant)	2006	CRURRS (NRRI)	CVRC, Uttar Pradesh, Bihar, Jharkhand, Assam	Irrigated	120-125 days; intermediate height (95-110 cm); SB; yield, 4.5-5 tha^{-1}; resistant to rice blast; moderately resistant to brown spot and gall midge
2	Anjali (RR 347-166)	Sneha / RR 149-1129	2002	CRURRS (NRRI)	CVRC, Uttar Pradesh, Bihar, Jharkhand, Assam, Tripura	Rainfed upland	90-95 days; intermediate height; SB; yield, 3.5 tha^{-1} in favourable uplands; moderately resistant to rice blast and brown spot
3	Annada (CR 222-MW-10)	MTU-15 / Waikoku	1987	NRRI	CVRC, Orissa, Madhya Pradesh, Manipur, Meghalaya, Nagaland, Goa	Rainfed upland and irrigated (early)	105-110 days; intermediate height; SB; yield, 4.75 tha^{-1}; moderately resistant to rice blast; sheath blight
4	Apo (IR55423-01)	UPLRi 5/ IR 12979-24-1 (BROWN)	2007	IRRI	Philippines	Aerobic	115-120 days; intermediate height (105-110 cm); yield, 3.9 tha^{-1} under aerobic condition; resistant to bacterial leaf blight; early vigour; drought tolerant
5	ASD16	ADT 31/ Co 39	1986	TNAU	CVRC, All India	Saline soils	115 days; intermediate height (93 cm); SB; yield, 5.6 tha^{-1}; salinity tolerant; moderately resistant to brown plant hopper and sheath rot
6	Bala* (CR 42-38-173)	N 22/ T(N) 1	1970	NRRI	CVRC, All India	Rainfed upland	105 days; semi-dwarf; SB; drought tolerant; moderately resistant to rice blast
7	CR Dhan 103 (CRR 451-1-B-2-1)	Vandana/ IR 64	2014	CRURRS (NRRI)	Jharkhand (yet to be notified)	Rainfed upland	95 days; intermediate height; LS; yield, 2.5-3.0 tha^{-1}; drought tolerant; resistant to blast; moderately resistant to brown spot
8	CR Dhan 40 (CRR 383-22)	N 22/ RR 20-5	2008	CRURRS (NRRI)	CVRC, Jharkhand Maharashtra	Upland and irrigated (early)	100 days; SB; yield, 3.0 -3.5 tha^{-1}; drought tolerant; resistant to gall midge; moderately resistant to rice blast, brown spot, sheath blight and leaf folder
9	Dular	Dumai/ Larkoch	1948		West Bengal, India	Rainfed upland	100-105 days; intermediate height (105 cm); LB; yield, 1.8-2.0 tha^{-1}; drought tolerance associated with greater root length and root density (Henry et al., 2011)
10	Heera (CR 544-1-2)	CR 404-48/ CR 289-1208	1991	NRRI	SVRC, Odisha	Rainfed upland	70-75 days; semi-dwarf (70-75 cm); LB; yield, 2.5-3.5 tha^{-1}; resistant to rice blast, rice tungro and gall midge
11	Kalinga III (CR-237-1)	AC-540/ Ratna	1983	NRRI	SVRC, Odisha, Gujrat	Rainfed upland	75-80 days; intermediate height; LS; yield, 2.75-3.0 tha^{-1}; cold tolerant; moderately resistant to brown spot.
12	Nagina 22 (N22)	Selection from Rajbhog	1978		Uttar Pradesh	Rainfed upland	90 days; intermediate height (110 cm); SB; drought and heat tolerant (Satake & Yoshida, 1978; Jagadish et al., 2008)

13	Poornima (IET-12284)	Poorva/ IR-8608-298	1997	IGKV	SVRC, Madhya Pradesh, Chhattisgarh	Rainfed upland	110 days; intermediate height (90-100 cm); LS; yield, 3.5- 4.0 tha^{-1}
14	Rasi (IET1444/ RPCB 849)	T(N)1/ Co 29	1977		CVRC, Andhra Pradesh, Orissa, Madhya Pradesh, Maharashtra, Tamil Nadu, Uttar Pradesh	Rainfed upland	115 days; intermediate height (90-95 cm); MB; yield, 3.0-3.5 tha^{-1}; resistant to rice blast; moderately resistant to rice tungro virus
15	Sadabahar (CR 306-37-13)	BRRI SAIL/ IR 10181-58 -3-1	2004	CRURRS (NRRI)	SVRC, Jharkhand	Rainfed upland	105 days; LB; yield, 3.2 tha^{-1}; moderately resistant to sheath blight.
16	Sahbhagi Dhan (IR74371-70-1-1-CRR-1)	IR55419-04*2/ Way Rarem	2009	CRURRS (NRRI)	CVRC, Orissa, Jharkhand	Drought-prone areas	105 days; LB; yield, 3.8-4.5 tha^{-1} and 1.0-2.0 tha^{-1} under moderate and severe drought stress, respectively; resistant to leaf blast; moderately resistant to brown spot, sheath rot, sheath blight and leaf folder
17	Sattari (CRM 13-3241)	CR 113 (NSJ-200/ Padma) mutant	1983	NRRI	CVRC, Orissa, West Bengal	Rainfed upland	72-75 days; semi-dwarf (65-70 cm); SB; yield, 2.5-3.0 tha^{-1}; being early often escapes diseases and pests
18	Shusk Samrat (NDR 1045-2)	C 1064-5/ Kalakeri// IR 54	2006	NDUAT	CVRC, Bihar, Orissa, UP	Rainfed upland	100-105 days; intermediate height; LB; yield, 3.0-3.5 tha^{-1}; moderately resistance to brown spot, sheath blight and sheath rot
19	Swarna (MTU 7029)	Vasista/ Mahsuri	1982	ARS, Maruteru	SVRC, Andhra Pradesh (Later notified for All India)	Rainfed Lowland	150 days; semi-dwarf; MS; yield, 6.5 tha^{-1}; Wide adaptability; Seed dormancy; High yielding low input response areas; a mega variety.
20	UPLRI7	C 22/IR26//C22/OS4	1970	UPLB	Philippines	Rainfed upland	110-115 days, intermediate height (95-100 cm); LB; yield, 3.5 tha-1 (aerobic condition); resistant to rice blast, early vigour, drought tolerant.
21	Vanaprabha (CR 289-1045-16)	ACR-12422/ ARC-12751	1988	NRRI	SVRC, Odisha	Rainfed upland	90 days; intermediate height (115-120 cm); LS; yield, 3.0-3.5 tha^{-1}; resistant to rice blast and rice tungro.
22	Vandana (RR-197-962)	C-22/ Kalakeri	2002	CRURRS (NRRI)	SVRC, Bihar, Jharkhand, Orissa	Rainfed upland	90 days; tall, LB; yield, 2.5-3.0 tha^{-1} and 3.5-4.5 tha^{-1} under direct seeded & transplanted conditions, respectively; weed competitive; drought tolerant, deep root system; doderately resistant to blast and brown spot.
23	Virendra (CRR 347-2)	Sneha/RR 149-1129	2006	CRURRS (NRRI)	CVRC, Odisha, Gujrat	Rainfed upland	95 days; SB; yield, 2.75 tha^{-1}; resistant to gall midge; moderately resistant to blast and brown spot.
24	Way Rarem	IR 9669/ B 981	1990-94		Indonesia	Rainfed upland	100-110 days; semi-dwarf (85-90 cm); yield, 2.97 tha^{-1}.
25	Bhutmuri	Landrace			West Bengal, India	Rainfed upland	95 days; intermediate height (110 cm); LB; drought ttolerant
26	AUS 257				West Bengal, India	Rainfed upland	90 days; intermediate height (110 cm); LB; drought tolerant
27	Black gora	Landrace			Jharkhand, India	Rainfed upland	95 days; intermediate height (120 cm); LB; high seedling vigour (Redoña & Mackill, 1996) and deep roots (Shrestha et al., 2014)

28	Kalakeri	Landrace			Odisha, India	Rainfed upland	90 days; intermediate height (100 cm); LB; drought tolerant; deep rooted
29	Kali aus	Landrace			India, Bangladesh	Rainfed upland	95 days; intermediate height (100 cm); LB; drought tolerant
30	Asanlaya	Landrace			West Bengal, India	Rainfed upland	95-100 days; intermediate height (110 cm); SB; drought tolerant
31	Lalnakanda-41	Landrace			Himachal Pradesh, India	Rainfed upland	100 days; intermediate height (110 cm); LB; drought tolerant
32	Moroberekan	Landrace			Ivory Coast, West Africa	Rainfed upland	135 days; tall (130 cm); MB; resistant to rice blast; drought tolerant; deep and thick root system.

[a]1-25, released varieties; 26-32, landraces

*Denotified

[b]Characteristics of released varieties recorded from: http://icar-nrri.in/released-varieties/ and http://drdpat.bih.nic.in

CRURRS: Central Rainfed Upland Rice Research Station, Hazaribag, Jharkhand, India; NRRI: ICAR-National Rice Research Institute, Cuttack, Odisha, India; IGKV: Indira Gandhi Krishi Viswavidyalaya, Raipur, Chhattisgarh, India; IRRI: International Rice Research Institute, Philippines; UPLB: University of Philippines, Los Baños, Philippines; VPKAS: ICAR-Vivekananda Parvatiya Krishi Anusandhan Sansthan, Almora, Uttarakhand, India; ARS, Maruteru: Regional Agricultural Research Station, Acharya N G Ranga Agricultural University, Andhra Pradesh, India; TNAU: Tamil Nadu Agricultural University, Coimbatore, TN, India; NDUAT: Narendra Deva University of Agriculture and Technology, Faizabad, Uttar Pradesh, India

Semi-dwarf: <90 cm; intermediate: 90-125 cm; tall: >125 cm (IRRI, 2013)

SB: short-bold; MB: medium-bold; LB: long-bold; LS: long-slender

References

IRRI (International Rice Research Institute) (2013) Standard evaluation system for rice (SES), 5th edn. International Rice Research Institute, Los Baños

Jagadish, S. V. K., Muthurajan, R., Oane, R., Wheeler, T R., Heuer, S., Bennett, J., & Craufurd, P. Q. (2008). Physiological and proteomic approaches to address heat tolerance during anthesis in rice (*Oryza sativa* L.). *Journal of Experimental Botany, 61,* 143-156.

Redoña, E. D., & Mackill, D. J. (1996). Mapping quantitative trait loci for seedling vigor in rice using RFLPs. *Theoretical and Applied Genetics, 92,* 395-402.

Satake, T., & Yoshida, S. (1978). High Temperature-Induced Sterility in Indica Rices at Flowering. *Japanese Journal of Crop Science, 47*, 6-17.

Shrestha, R., Al-Shugeairy, Z., Al-Ogaidi, F., Munasinghe, M., Radermacher, M., Vandenhirtz, J. & Price, A. H. (2014), Comparing simple root phenotyping methods on a core set of rice genotypes. *Plant Biology Journal, 16,* 632-642.

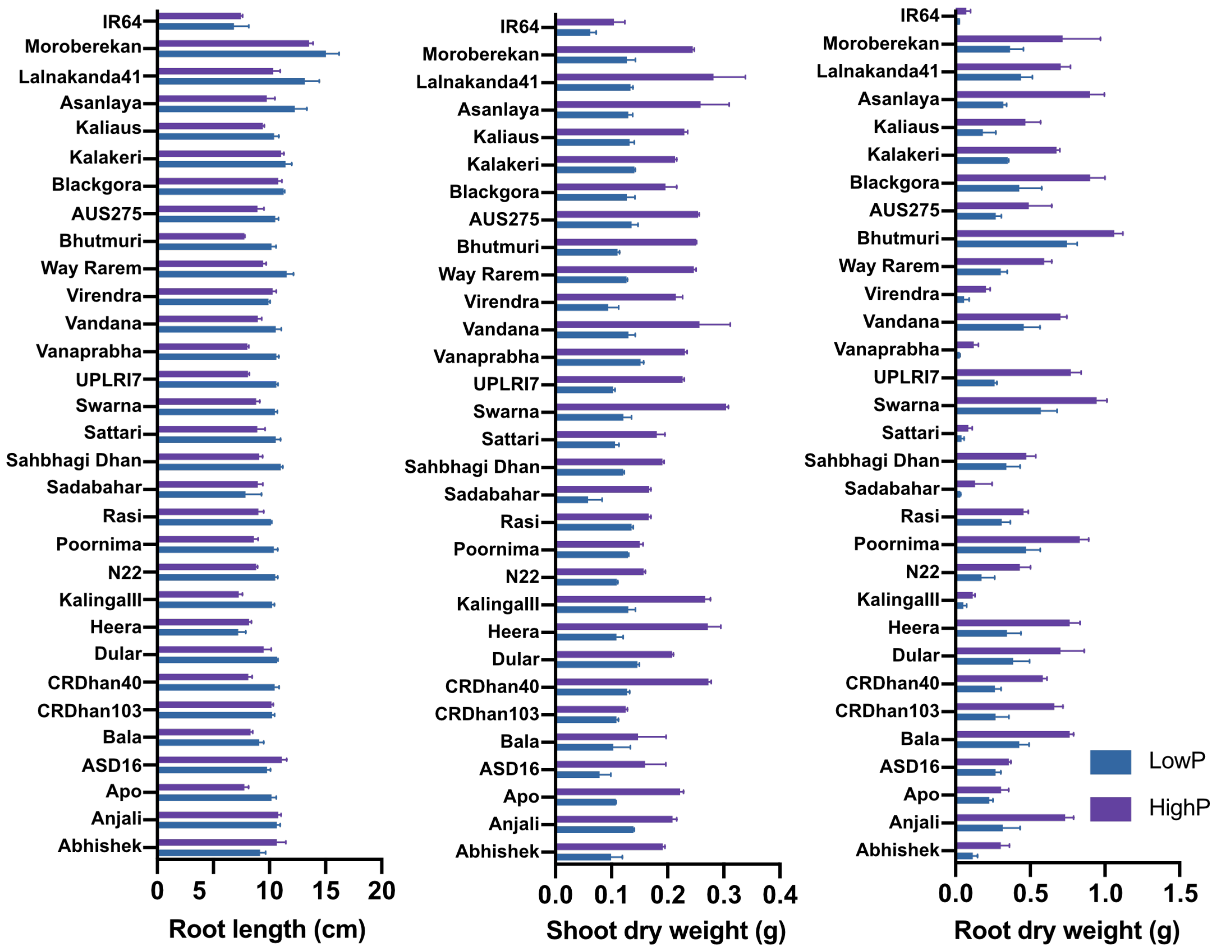

Fig. S1 Effect of genotype and phosphorus treatment on root and shoot traits. Values are mean of three observations ± SD. The figure was edited for presentation using Affinity Designer 1.9.3 (https://affinity.serif.com/en-gb/).
